# A Telehealth Home-Based Exercise Program for Community-Dwelling Older People with Dementia in Indonesia: A Feasibility Study

**DOI:** 10.3390/ijerph20043397

**Published:** 2023-02-15

**Authors:** Yulisna Mutia Sari, Elissa Burton, Den-Ching A. Lee, Keith D. Hill

**Affiliations:** 1 Rehabilitation, Ageing and Independent Living (RAIL) Research Centre, School of Primary and Allied Health Care, Monash University, Melbourne 3800, Australia; 2 Curtin School of Allied Health, Curtin University, Perth 6102, Australia; 3 enAble Institute, Curtin University, Perth 6845, Australia; 4 National Centre for Healthy Ageing, Monash University and Peninsula Health, Melbourne 3199, Australia

**Keywords:** dementia, exercise, Indonesia, older adults, telehealth

## Abstract

(1) Background: This study aimed to evaluate the feasibility of a telehealth home-based exercise program for older people with dementia living in Indonesia with support from their informal carers. (2) Methods: Pre–post intervention single group study with three assessment time-points (baseline, 12 and 18 weeks). Participants with dementia underwent a 12-week physiotherapist-delivered telehealth exercise program, with informal carer supervision between supervised online sessions, and continued the exercises for a further six weeks without physiotherapist online supervision. (3) Results: Thirty dyads of older people with dementia and their informal carers were recruited; four (13.3%) withdrew across the 12-week intervention and one (3.3%) in the 6-week self-maintenance period. Median adherence was 84.1% (IQR [25, 75] = 17.1) during the 12-week intervention, and 66.7% (IQR [25, 75] = 16.7) in the self-maintenance period. No falls/adverse events were reported. Physical activity level, some aspects of function and disability, health-related benefits of exercise, exercise enjoyment and quality of life of older people with dementia improved significantly at 12 and 18 weeks. (4) Conclusions: The telehealth exercise program is feasible and safe and may have benefits for the health outcomes of community-living older people with dementia in Indonesia. Additional strategies are necessary to enhance longer-term adherence to the program.

## 1. Introduction

Dementia is an increasingly important public health concern as it can lead to major disability and dependency among older people as well as a heavy physical, psychological and economic burden on family and carers, health and social care systems [1,2]. As the aging population increases, the number of people living with dementia worldwide is growing rapidly [3]. Much of the rapid growth in aging populations is occurring in low- and middle-income countries in Asia [4]. This creates challenges in providing health care services that focus on older populations, and particularly dementia care, in these low- and middle-income Asian countries, such as Indonesia [5].

Engaging in regular physical activity is beneficial for older people with dementia, with benefits including improved physical health, function, independence, psychological health and wellbeing among older people with dementia [6,7,8], improved cognition [9] and reduced impact on their carer [10]. There is also growing evidence that exercise programs (a structured form of physical activity) are feasible in people with dementia and can achieve similar health benefits for those with mild and moderate severity of dementia [11,12]. A number of different types of exercise have been shown to be feasible and safe for people with dementia, including resistance training, cardiovascular fitness exercises, balance exercises, and multi-modal (combined) approaches; and have been successfully delivered in home-based (individual), community or group settings [9,13]. However, people with dementia are generally less likely to participate in regular physical activity compared with their peers without dementia [14,15].

Barriers to increasing physical activity levels in people with dementia have been identified: some from the perspectives of people with dementia and their carers, e.g., memory impairment, lack of transportation, lack of time (fitting in fixed times for physical activity around multiple other appointments), limited support from carers, lack of knowledge of how to exercise and fear of injury (risk of falling during physical activity); and some from the perspective of health professionals or service providers, e.g., limited resources (insufficient staffing and suitable programs) [15,16,17]. These barriers to undertaking physical activities for people with dementia have been further exacerbated by the on-going global coronavirus (COVID-19) pandemic. An important and less recognized, indirect consequence of the pandemic is that it has left many older people, including those with dementia, who are vulnerable to greater risk for severe illness from infection [18] restricted to home, with reduced opportunities and resources available to support their ongoing participation in physical activity or exercise in the community [19]. Ongoing impacts of this is the potential for increased risk for falls, a decline in their physical function and negative impact on the cognitive and neuropsychiatric symptoms of older people with dementia [20]. Therefore, an intervention designed to increase physical activity levels for people with dementia and address these challenges is necessary. Exercising at home has been suggested to minimize the health consequences of sedentary behavior during the pandemic [21]. Previous studies have reported that delivering exercise interventions via telehealth are feasible for increasing physical activity levels in older people with dementia and their carers in developed countries [22]. This approach may have potential benefits since it requires no travel commitment, less cost and offers support for carers’ engagement to enhance participation by the person with dementia.

Exercise studies for people with dementia have been conducted in several developed countries [6,8,11,23], but very few in developing countries such as Indonesia [24], with beneficial outcomes including improved physical and cognitive function being reported. However, more research is needed, particularly in developing countries, as there may be local factors in these countries that limit successful translation of these programs in the contexts of culture, environment, knowledge/preferences of consumers, and health care systems. For example, there were cross-cultural issues affecting research translation identified in reviews of other health conditions [25], including falls [26]. In addition, a telehealth-delivered exercise intervention may have potential benefits in improving psychological and physical health and wellbeing outcomes of older people in developed countries [22,27]; however, the feasibility and safety of a telehealth-delivered exercise intervention to older people with dementia in developing countries, such as Indonesia, have yet to be established. Similar to other Asian countries with strong family and cultural values, in Indonesian culture, physical activity or exercise is equated to participating in activities of daily living, such as showering, cleaning and preparing meals, and being old often means older people should rest and not take part in physical activity or exercise [28]. Therefore, the aims of this study were to investigate in Indonesia (i) the feasibility (including demand, implementation, practicality (including safety), adaptation, acceptability and efficacy) of delivering a 12-week telehealth exercise program by a physiotherapist with informal carer supervision of the exercise program, and a subsequent self-maintenance six- week period without physiotherapist supervision (only with carer supervision) (week 13–18, (ii) the preliminary effects of the telehealth-delivered exercise program on physical activity level, lower limb function and disability, health-related benefits of exercise, fear of falls, exercise enjoyment, quality of life, and behavior (neuropsychiatric symptoms) for older people with dementia, as well as the impact on their informal carer(s), and (iii) the sustainability of exercise participation in the 6-week self-maintenance period post-intervention.

## 2. Materials and Methods

### 2.1. Design

A single group pre–post intervention study design with three assessment time-points: (1) baseline (denoted as T_0_ pre-intervention), (2) 12th week (T_1_ at intervention completion) and (3) 18th week (T_2_ at 6-week self-maintenance post-intervention) was conducted.

### 2.2. Study Participants

Study participants were older people with dementia and their informal carers (family members and/or paid carers; a paid carer in this study is someone who is paid and lives with a person with dementia to look after them at home). The inclusion criteria for people with dementia were (a) community-dwelling in Indonesia, (b) aged ≥ 60 years, (c) diagnosed with dementia (any type) with mild to moderate severity (Telephone Mini Mental State Examination (T-MMSE) score of 8–23) [29], (d) able to understand and follow instructions for exercises in the Indonesian language, (e) able to walk independently or with supervision (with or without a gait aid), (g) not having other major neurological history or medical conditions preventing participation in exercise, (h) having a carer to assist in the supervision of exercises, and (i) having a computer/tablet and internet connection. People with dementia who had unstable medical conditions, or speech and hearing impairments that may impede participation in the telehealth-delivered exercise program were excluded. Inclusion criteria for carers were (a) aged ≥ 18 years, (b) availability to participate in the online exercise sessions and supervise the person with dementia to exercise in between the online sessions, (c) familiarity with the participant’s medical conditions, and (d) ability to communicate with the person with dementia and the researcher in the Indonesian language. Carers were excluded if they had conditions that prevented them from participating in the program (e.g., physical and/or mental limitations).

### 2.3. Sample Size

A sample size of 30 dyads of older people with dementia and their carers (i.e., 30 people living with dementia and 30 carers in total) was deemed sufficient to evaluate the feasibility of delivering the intervention, and determine the demand, implementation, practicality (including safety), adaptation and acceptability of the exercise program, based on the median sample sizes reported for this type of study in an audit of pilot and feasibility trials in the United Kingdom Clinical Research Network [30]. This study was not powered for the efficacy testing (described below), although data were collected to inform future definitive studies that may investigate these outcomes in Indonesia.

### 2.4. Recruitment Process

Approval for the study was obtained from the Monash University Human Research Ethics Committee (MUHREC) (Project ID: 22163) and Health Research Ethics Committee Universitas Muhammadiyah Surakarta (No. 2856/B.1/KEPK-FKUMS/III/2020). Older adults with dementia and their carers were recruited through the Alzheimer’s Indonesia Association newsletter, mailouts to the Alzheimer’s Indonesia members (i.e., people with dementia and their carers), advertisements in local newspapers, local dementia and carer support groups, hospitals, community services for older people and social media. People who were interested were asked to contact the researcher by email or phone for further information about the study. All participants with dementia and their carers were provided with an Explanatory Statement and the opportunity to ask questions. If they were willing to participate, they were asked to sign the written consent form and return it by email (with scan or photo of signed form) or post prior to commencing the study. Carers were asked to provide written consent on behalf of the participants with dementia to participate, if the person with dementia was unable to provide informed consent (all participants with dementia were assessed for their capacity to give consent using the Participant Cognitive Capacity Checklist) [31].

### 2.5. Data Collection

People living with dementia and their carers were screened for eligibility. Eligible dyads completed the study consent forms prior to the initial assessment session taking place. An initial online assessment via video conferencing using the Zoom application was undertaken to obtain information on: (1) the person with dementia and carer’s demographics, (2) history of falls for the preceding 12 months for the participants with dementia, (3) physical status; and (4) secondary (efficacy related) outcomes including (a) physical activity level, (b) lower limb function and disability, (c) health related benefits of exercise, (d) fear of falls, (e) exercise enjoyment, (f) quality of life, (g) behavior (neuropsychiatric symptoms) and (h) perceived impact on their carer/s (see details of assessment tools below). The assessment of these secondary outcomes was repeated at 12 weeks (at the end of the intervention phase), and 18 weeks (at 6 weeks post-intervention phase) to provide preliminary data on sustainability of exercise participation beyond the intervention period. Data were collected from the dyads using self-reported scales and questionnaires. All measures for people living with dementia were obtained from people with dementia (if possible) and/or their carers (i.e., reporting from the carer’s perspective) as required. The scales and questionnaires were sent to the participants via email or post prior to study commencement. An exercise diary was used to document adherence to the program by participants with the assistance of their carer (i.e., collecting data on times/week, type of exercise, sets and repetitions of exercises undertaken).

### 2.6. Intervention

#### 2.6.1. Orientation Session

Participating dyads attended an on-line orientation session conducted within the baseline assessment session led by the researcher (YMS). In this session, the researcher explained and demonstrated the Zoom software, tested the camera and sound for performing the exercise program, set the device distance and environment for performing exercise, and explained about safe performance on undertaking the exercises and provided time for questions.

#### 2.6.2. Exercise Sessions

Participants undertook an individualized home-based exercise intervention for 12 weeks, with four exercise sessions (online visits, in real-time) delivered via video conference by the researcher (YMS, who is also a physiotherapist) in this period. The dyads were instructed to continue the home exercise intervention for 5 days per week, 20–30 min each day in between the scheduled online visits (this dosage aligns with the recommendations for people with dementia in the systematic review and meta-analysis by Lam et al.) [8]. The exercises included warm up, balance, resistance and walking exercises, with the physiotherapist aiming for moderate-intensity exercise level, in terms of exercise demands and difficulty for each participant (e.g., by changing the repetitions, load, and foot position). The four online (visit) sessions were scheduled at weeks 1, 2, 6 and 10 during the 12-week intervention phase (Table 1). This exercise program was modified from the Otago exercise program (an exercise program for the prevention of falls) (https://www.livestronger.org.nz/assets/Uploads/acc1162-otago-exercise-manual.pdf, accessed on 2 December 2022) to ensure safety of the dyads when participating in the online visits and home exercise (available from the authors on request). The Otago program, which is usually delivered face-to-face, has resulted in benefits in improving strength, balance, endurance and also reduces falls among older people living in the community [32,33] including older people with cognitive impairment and dementia [11]. The version of the Otago program as delivered by a physiotherapist in the homes of people living with dementia reported by Suttanon et al. [11] was adapted for this study (available from the authors on request). As well as the mode of delivery being modified (to telehealth delivery), other modifications made were exercising with a stable chair or bench on either side that could be used for steadying if required, and standing with a wider distance between feet was also applied as required to ensure the participant’s safety. Exercise participation was encouraged and monitored for safety by the carer, who also received training by the researcher (adapting the approach and resources used by Suttanon et al. [11]) in the first online exercise visit.

Prior to the first online exercise session (after the assessment session), the researcher selected the appropriate exercises from the modified Otago Exercise program, tailored to the physical performance and needs of the person with dementia. These were performed at the first online exercise session, in view of the researcher using the video conference camera, with speaker on so that the participant with dementia and carer could hear the instructions of the researcher, and the researcher could observe and give instructions for the safe performance of the exercise intervention. Also at this online visit, the researcher ensured that the carer understood and was confident to be able to supervise the exercise sessions independently between the physiotherapist-supervised online visits, by providing practice opportunities and feedback and support. At each of the next three online visits, the researcher monitored and progressed the exercise intervention (to encourage improvement) where required and answered any questions. Between the four online visits, the carer was instructed to supervise the exercise intervention for the duration and frequency listed above. Each participating dyad received an exercise booklet (sent via email) that explained the selected exercises in simple terms and with pictures after the first session to support ongoing participation in the exercises. The booklet was updated when changes to the exercise selection occurred. The dyads were also provided with YouTube video links of the prescribed exercises, to facilitate correct exercise between the online visits. After the 12-week intervention, the dyads were asked to continue with the exercise for an additional six weeks without the researcher’s online supervision (self-maintenance phase).

Safety of participants was supported through supervision and observation of their exercise performance through the online visits, and if necessary, the researcher would provide instruction to stop an exercise if the participant appeared unsteady or complained of pain. Participants were instructed to exercise in a safe environment within their home, involving exercising with a stable chair or bench on either side that could be used for steadying if required. In prescribing the exercises, modifications (e.g., standing with a wider distance between feet) were applied as required to ensure the participant was safe. If an unanticipated event occurred during the sessions supervised by the family carers (e.g., feeling unwell, dizziness, or fall), the participant and the carer were asked to stop all exercises, and to contact the researcher to discuss the event and management, and if needed, to seek relevant medical assistance (e.g., local doctor or Emergency Department).

#### 2.6.3. Check-Up/Support Session

The researcher contacted the dyads four times via videoconference calls at week 3, 5, 8 and 11 scheduled in between the four online visits (Table 1) to check up and provide support for the dyads for the maintenance of exercise. The dyads were instructed to contact the researcher if they had any concerns regarding the exercises or to report an adverse event that may have occurred during the study.

### 2.7. Outcomes Measures

The outcomes measures were the feasibility measures, evaluated using Bowen et al.’s six domains including demand, implementation, practicality (including safety), acceptability, adaptation and efficacy testing [34].

#### 2.7.1. Demand

Demand was defined as the number of participants who (1) enquired about the program, (2) were recruited into the program, and (3) completed the 12- and 18-week programs.

#### 2.7.2. Implementation

Implementation was defined as the extent the program was successfully delivered as planned. Exercise adherence rate (the number and proportion of scheduled sessions undertaken by the participants) and reasons for cancelling recommended exercise sessions were documented. Full program adherence (100%) was defined as a participant undertaking the home-based exercise program five times a week for 12-week intervention phase (total of 60 recommended exercise sessions across 12 weeks) and additional 6 weeks of self-maintenance phase (total of 30 recommended exercise session across 6 weeks). Adherence was calculated as the percentage of recommended exercise sessions completed (an exercise diary was provided to participants by email or post, for them to complete each day they exercised, to provide this information, which was emailed or posted to the researcher each month). Overall program adherence was calculated by dividing the total mean adherence across all participants by the number of participants. In addition, the median number of exercises conducted per session was documented. This intervention was considered feasible if (i) the mean adherence rate was ≥70% [22], and (ii) ≤30% participants withdrew from the study over the 12-week component of the study (drop-out rate) [22].

#### 2.7.3. Practicality (Including Safety)

Practicality refers to whether participants were able to perform the telehealth-delivered exercises supervised by the physiotherapist and also without supervision of the physiotherapist (with only carer supervision). In addition, safety of the program, defined as the frequency and nature of adverse events, was also evaluated. An adverse event in this study was any unexpected event related to the intervention with a potential impact on participants, including a fall, emergency events (e.g., cardiac arrest, angina), injury or pain sustained from the exercises. This exercise program was considered safe if ≤10% of participants reported an adverse event [22]. For this study, a fall was defined as “an event which results in a person coming to rest inadvertently on the ground or floor or other lower level” [35]. Data on the number, cause, location, related injuries and nature of any fall were collected prospectively using a falls diary and verified during the video conference meetings (this included any falls, including if a fall occurred during the exercise program, as well as falls at any other time).

#### 2.7.4. Acceptability

This area of feasibility reported participant’s satisfaction of the program. The participants’ perceptions about participating in the telehealth program were investigated but will be reported separately (a qualitative study by Sari et al., manuscript under review [36]).

#### 2.7.5. Adaptation

Adaptation describes any changes made in the exercise program to meet the needs of older people with dementia in Indonesia and to tailor the new method (telehealth using videoconferencing) and to ensure the participant’s safety. This exercise program was individualized and tailored based on participants’ physical performance, assessment findings, and observation of participant’s initial attempts at selected exercises determined as suitable by the physiotherapist. The exercise level was gradually progressed over time for participants as they improved their ability to complete the program’s current exercises.

#### 2.7.6. Efficacy Testing

Based on Bowen’s feasibility framework, efficacy testing is the effects of the program and whether these were sustained after completion of the supervised intervention [34]. Various measures were evaluated at baseline, week 12 and week 18. Assessment measures were undertaken on-line at the three-assessment time-points by the same physiotherapist (YMS), who was also the physiotherapist conducting the online exercise sessions.

Secondary outcomes were used to provide a preliminary evaluation of the efficacy of the program. Because of the telehealth nature of the interactions (including assessments), efficacy measures were limited to validated questionnaire/self-report-based tools. Efficacy outcomes included:

1. Physical activity level—assessed using the Physical Activity Scale for the Elderly (PASE) [37]. The PASE is a 12-item assessment tool that combines several types of physical activity including household, leisure, and occupational activity over a seven-day period. The range of scores is 0–400, and higher scores indicate higher levels of physical activity [37].
2. Function and disability—assessed using the Late-Life Function and Disability Instrument (LLFDI) [38]. The LLFDI is an evaluative outcome measure designed to assess function (ability to perform activities in daily routines) and disability (performance in socially defined tasks) for community-dwelling older people. The function component comprises 32 items and an additional eight device items (for those who use canes and walkers) within three domains (Upper Extremity Functioning, Basic Lower Extremity Functioning and Advanced Lower Extremity Functioning). Both raw scores of function and disability components were transformed to a scaled score (0–100), with higher scores indicating higher level of function. The disability component comprises 16 items over two dimensions (frequency and limitation); The frequency dimension comprises a Social Role domain and Personal Role domain and the limitation dimension comprises the Instrumental Role domain and Management Role domain. Higher scores in frequency indicate high levels in frequency of participating in life tasks and higher score in limitation signify higher levels in capability of participating in life tasks [38].
3. Health-related benefits of exercise—assessed using the Vitality Plus Scale [39], a 10-item self-reported Likert scale in which participants rated health domains including sleep, bodily pain, energy, bowel function and appetite with a maximum score of 50 (each item is scored on a 1–5 scale). Higher scores indicate better perceived health [39].
4. Fear of falls—assessed using the Iconographical Falls Efficacy Scale (Icon-FES). The Icon-FES is an innovative and valid fear of falling measurement for older people with cognitive impairment [40]. This scale uses pictures to describe the range of situations and activities. Scores range from 10–40, and higher scores indicate greater fear of falls [41].
5. Exercise enjoyment—assessed using the 8-item Physical Activity Enjoyment Scale (PACES) [42]. The PACES scale evaluated how the participant felt about physical activity that they had been doing, using a 7-point Likert scale. Scores range from 8–56. Higher scores indicate increased levels of enjoyment from participating in the physical activity.
6. Quality of Life—assessed using the Quality of Life in Alzheimer’s Disease (QOL-AD) tool [43]. This scale is designed to assess the quality of life of people with dementia from both people with dementia and their carer’s perspective. It includes 13 items measured (range from 13–52) using a Likert scale, and higher scores indicate better quality of life [43].
7. Behavior (neuropsychiatric symptoms)—assessed using the Neuropsychiatric Inventory Questionnaire (NPI-Q). The NPI-Q is a valid and reliable questionnaire to assess neuropsychiatric symptomatology in dementia that includes 12 domains of neuropsychiatry, with scores ranging from 0–36 [44]. This screening questionnaire also provides the Caregiver Distress Scale for identifying the impact of the behaviors on the carer with scores ranging from 0–60. Higher scores indicate higher level of severity of neuropsychiatric symptoms and the distress experienced by the carer [44].
8. Impact on informal carer—assessed using the Zarit caregiver burden scale. The Zarit caregiver burden scale is widely used and comprises a 22-item questionnaire with a maximum score of 88; higher scores indicate higher burden [45].

All measures used in this study have been shown to be valid and reliable for older people and older people with dementia [15,38,39,40,42,43,44,46,47,48,49].

### 2.8. Data Analysis

Analyses were undertaken using Statistical Package for the Social Sciences (SPSS) (version 26). Analyses of feasibility were descriptive or based on estimates with 95% Confidence Interval (CI) values. Continuous variables were presented as means with standard deviations (SD) or medians with interquartile ranges (IQR) depending on normality of distribution. Analyses were conducted on an intention-to-treat basis. Multiple imputation of missing data (missing at random) was used to manage missing data due to participants dropping out during the study duration. For the efficacy testing component, to evaluate the preliminary effects of the exercise program, one-way repeated measures analysis of variance (1-way RM ANOVA) was conducted to evaluate change across the three time points (baseline, 12 weeks, 18 weeks). The post-hoc least significant difference (LSD) test was used to determine pairwise significance between time points. The *p* value for analyses was set at <0.05. However, to adjust for multiple comparisons where several measures were assessing a similar outcome domain, Bonferroni adjustments were made to the *p* value (for example, for the subcomponents of the LLDFI Function, three measures were analyzed, the *p* value for these three analyses was adjusted to 0.05/3 = 0.0167); and for both the Disability Frequency and Disability Limitation components of the LLDFI, each of which had two measures analyzed, the *p* values for each measure was adjusted to 0.05/2 = 0.025. Cohen’s d Effect Sizes were calculated for all significant outcomes, and reported as small (d = 0.20–0.49), moderate (d = 0.50–0.79) or large (d ≥ 0.80) [50].

## 3. Results

### 3.1. Participants and Demand

**Figure 1:**
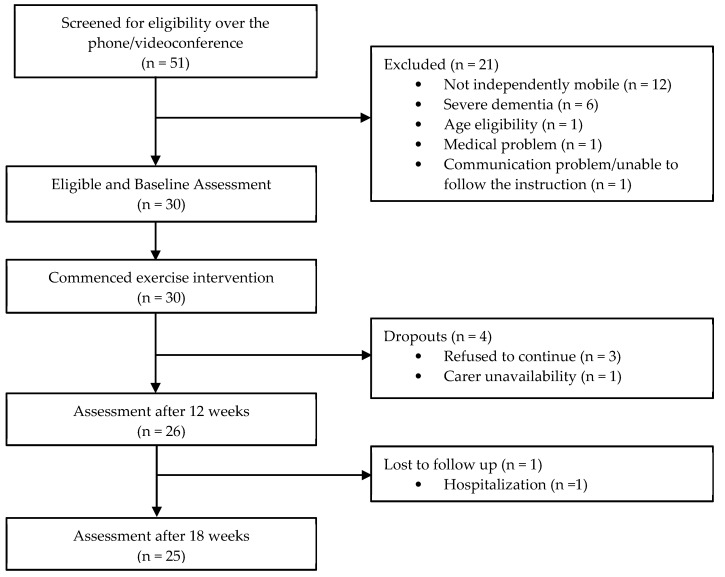
Flow diagram of recruitment and attrition.

Recruitment commenced in January 2021 and data collection ceased in July 2022. Thirty dyads of people with dementia and their carers were recruited online from across Indonesia (Figure 1). The percentage of participants who completed the 12-week exercise intervention was 86.7% (N = 26), and 83.3% (n = 25) of participants completed the additional six-week self-maintenance period. Characteristics of participants are reported in Table 2. Most participants with dementia were females with an average age of 71.5 (SD 8.8) years. The most common dementia type was Alzheimer’s Disease (73%), average duration post dementia diagnosis was 2.1 years (SD 1.2), and each participant with dementia had at least one medical condition in addition to their dementia. Most participants with dementia (83%) lived with family carers, and 10 participants with dementia (33.3%) had a paid carer. Most of the family carers and paid carers were female (80% and 90% respectively), and many of the family carers were a child of the person with dementia (83.3%).

### 3.2. Implementation

All planned telehealth-supervised exercise sessions and check-up/support sessions were completed within 12 weeks for all participants. The total number of participants who withdrew was four (13.3%) across the 12-week intervention and one (3.3%) in the 6-week self-maintenance period post intervention. Reasons for withdrawing included the participants with dementia choosing not to continue with the program, carer unavailability and hospitalization of person with dementia (flow diagram of recruitment and attrition is reported in Figure 1). The median number of exercises undertaken per session, the number of exercise sessions undertaken and median adherence (%) to 12 weeks with physiotherapist’s supervision and the 6-week self-maintenance (i.e., weeks 13–18) phase of the exercise program are reported in Table 3. Reasons for not undertaking the exercises in this exercise program included mood or refusal to do the exercise, illness or being unwell, other commitments and travel by the participant with dementia, carer unavailability and carer was overwhelmed managing competing issues related to the person with dementia they looked after.

### 3.3. Practicality (Including Safety)

One physiotherapist (YMS) supervised the exercise program through videoconferencing (telehealth). All participants with dementia were able to perform the exercises in the supervised sessions conducted by the physiotherapist and also with carer’s supervision (without the physiotherapist). Three participants (10%) reported some discomfort (e.g., muscle or joint soreness) after the first session or pain due to aggravating a pre-existing condition (e.g., arthritis). The intensity and movement patterns of the exercise were modified if discomfort or pain were reported. No falls or other adverse events occurred during the 12-week intervention nor the additional 6-week self-maintenance (to 18 weeks) phase of the exercise program.

### 3.4. Acceptability

Qualitative outcomes exploring participants’ perceptions and satisfaction with the program revealed that participants perceived positive health and psychological benefits and were satisfied with the program (further detail from semi-structured interviews is reported in a separate paper, in preparation).

### 3.5. Adaptation

The adjustments made prior to this study to the successful face-to-face method reported by Suttanon et al. [11] (see Section 2) were accepted well by participants. There was no further adaptation required during this study.

### 3.6. Efficacy Testing

Results of the preliminary effects of the exercise intervention are reported in Table 4. Several outcomes had statistically significant improvements over time in the one-way ANOVA analyses (using Bonferroni adjusted *p*-values where appropriate), including the PASE, LLFDI function component (basic lower extremity domain), LLFDI–disability component total (frequency dimension), LLFDI–disability component (personal role domain), Vitality plus scale, 8-item PACES and QOL-AD (for both people with dementia and carer components). In most cases where significant overall ANOVA *p* values were obtained, the post hoc analyses identified a significant difference between baseline and both the 12- and 18-week scores (allowing for Bonferroni adjustment), but no significant differences between the 12-week and 18-week scores were identified. No significant changes were observed for the remaining outcome measures. Effect sizes for the significant changes from baseline to 12 weeks ranged from 0.60 (PASE) to 2.33 (8-item PACES); and from baseline to 18 weeks ranged from 0.47 (LLFDI–disability component (instrumental role domain)) to 2.17 (8-item PACES) (Table 4).

Although several measures in this study show significant change over the intervention period, several important measures (e.g., Icon-FES and NPI-Q (severity and carer distress)) showed a trend of improvement but did not reach significance. Post-hoc analysis using the mean and SD data from this study using G*Power software (version 3.1) indicated a sample size of 76, 78 and 13 required for each group, respectively, for a power of 0.80 and *p* < 0.05 for the Icon-FES and NPI-Q (severity and carer distress) measures, respectively.

## 4. Discussion

Study findings suggest that a telehealth home-based exercise program delivered and progressed through intermittent videoconferencing by a physiotherapist, with carer supervision between the supervised online sessions over 12 weeks, is feasible and has benefits on physical activity level, some aspects of function (basic lower extremity domain) and disability (frequency dimension total and personal role domain), health-related benefits of exercise, exercise enjoyment and quality of life of older people with dementia living in the community in Indonesia. The program was deemed safe, with no adverse events or falls occurring. There was high adherence during the 12-week physiotherapy supervised component of the exercise program; however, this decreased in the subsequent six-week period when the online physiotherapist supervision ceased. The positive physical and health-related outcomes suggest that opportunities for greater use of telehealth-delivered exercise programs of this nature for older people with dementia living in the community warrant exploration. However, there is a need to incorporate improved approaches to support longer-term adherence to independent exercise (with carer supervision) without formal physiotherapist oversight (e.g., additional education resources).

This study took place during the height of the COVID-19 global pandemic, which had a significant impact in Indonesia, where this study was conducted [51] and elsewhere globally. During the pandemic, social isolation was an important public health requirement due to the ease of contracting COVID-19 and the risk posed to older people [52]. Many traditional opportunities for formal and informal physical activity/exercise were not able to be undertaken (for example, gymnasiums and exercise centers were closed) and many older people have been reluctant to resume regular physical activity [19], particularly involving interactions with other people/groups, since pandemic restrictions have eased. Many studies have reported reduced physical activity levels for older people during the pandemic, and many have not returned to pre-pandemic levels, which is likely to result in worsened mobility and increased risk of falling unless new opportunities to increase physical activity are introduced [53,54]. This telehealth-delivered exercise program provided a new opportunity for exercise to be initiated and continued within the home, with online assessment and supervision, despite the barriers imposed by the pandemic. The positive feasibility outcomes of the approach in our study, which was necessitated initially because of the timing of the pandemic with commencement of this study, is likely to be a suitable longer-term option beyond any remaining impacts of the pandemic. It may be useful for some older people who will have ongoing reluctance to resume interactions for physical activity participation that involve being away from home. In addition, the approach has considerable applicability for people with dementia and their carers living in regional and rural areas, where direct face-to-face access with physiotherapists to supervise this type of program may be limited.

The findings from this study have particular relevance to exercise for people with dementia in Indonesia and other developing countries that are on a rapid aging trajectory and that have some unique differences compared to developed countries in parts of the world other than Asia. These include cultural differences (e.g., beliefs about causes of health problems and how to treat these) [25], regular use of paid as well as informal (family) carers [55], and developing a health professional workforce with knowledge and skills to treat aging-related health problems such as dementia [56].

Few studies have evaluated telehealth as a delivery approach for exercise programs for older people with dementia living in the community to compare our results with. Ptomey et al. conducted a pilot trial of a group videoconferencing exercise program for older adults living with Alzheimer’s disease (n = 9 people with Alzheimer’s disease and caregiver dyads) for 12 weeks [22], and Dal Bello-Hass et al. investigated an exercise program where dyads (n = 2 people with dementia and caregiver dyads) came to telehealth sites to undertake telehealth exercise sessions and conducted a small short-term (4 weeks) study [57]. While these previous studies only report the attendance rate of the telehealth-delivered sessions without reporting the adherence rate of recommended exercise sessions being undertaken at home with carer supervision, together, these studies demonstrate that telehealth-delivered exercise programs are feasible in this population. There is also potential for use of telehealth approaches to address a range of other health problems for older people with dementia, for example, remote medication management [58]. Benefits of the telehealth approach for people living with dementia in the community include improved access to care (including those living away from metropolitan areas), and improved timeliness of access to and efficiency of services [59].

The adherence rate in this study was high (median of 84.1%) and similar to a previous face-to-face in-home exercise program for people with dementia with carer supervision by Suttanon et al. [11] (adherence rate of 83%). However, the high adherence during the 12-week program which included formal exercise and support sessions by a physiotherapist was not transferred to the self-maintenance duration (additional 6 weeks) without any physiotherapist online visits (median adherence rate of 66.7%). The main reasons for not undertaking the exercise sessions during the self-maintenance period in the previous study were refusal to exercise by participants with dementia, health conditions, and carers reporting substantial challenges managing other issues related to the person with dementia that they looked after. Overall, carers (family or paid) were able to supervise and encourage people with dementia to participate in a home exercise program with intermittent physiotherapist guidance through regular online visits, but it was more difficult to encourage them to participate when physiotherapist supervision ceased. Previous studies indicated that support from carers is a critical component for successful exercise program engagement by people with cognitive impairment/disability and dementia [11,60]. This present study also indicates the support of the physiotherapist is important not only for the person living with dementia but for their carer also. Providing tips to motivate carers to continue and how carers can motivate people with dementia to continue the exercise is important in the future programs.

The drop-out rate in this study was 13.3% during the 12-week program and 16.7% during the 18-week program, which is lower than in a previous face-to-face exercise study by Suttanon et al. (42%) [11] and the video-conferencing approach by Ptomey et al. (22%) [22]. The reasons for withdrawing from the exercise program in these other studies were refusal to continue, moving to care facility, hospitalizations and passing away of participants with dementia, and health condition of the carers, which has some similarity to the reasons for withdrawing in this study.

No falls or other adverse events occurred during this study. This aligns with previous physiotherapist delivered exercise programs for people with dementia living in community, with carer supervision [11] and a telehealth exercise program for people with Alzheimer’s disease [22]. This suggests that telehealth exercise programs with physiotherapist online visits and home-based exercise programs with carer supervision are safe to maintain physical and psychological health of older people living with dementia in the community.

This study was designed as a feasibility study. Given the relatively novel intervention approach with this clinical group (people with dementia), the sample size was determined relating to testing feasibility, rather than to determine effectiveness of the secondary (outcome) measures. It was anticipated that data relating to these outcomes could inform sample size calculation for a future randomized controlled trial. However, it must be noted that in this study, several measures did demonstrate significant improvement, particularly in the physiotherapist-supervised period, and most others were trending in the direction of improvement, despite not reaching significance. Most positive changes in outcomes were sustained in the self-maintenance 6-week period without physiotherapist online supervision, and despite the lower ongoing exercise participation, which is an encouraging outcome. Post hoc sample size analyses of several of the important measures not reaching significance in this study indicate that a definitive adequately powered trial should include an overall sample of 278 people with dementia, and their carers. Ideally, a future fully powered randomized controlled trial will strengthen confidence that this telehealth physical activity program can improve physical, psychological and wellbeing outcomes for people with dementia, and by using a hybrid effectiveness-implementation design, also identify key factors contributing to the outcomes.

There were several strengths to this study, including that this is the first study to provide evidence on feasibility of telehealth home-based exercise programs for older people with dementia living in the community in Indonesia. In addition, the efficacy testing component of the study highlighted the positive outcomes to inform future studies on physical activity level, some aspects of function and disability, health-related benefits of exercise, exercise enjoyment and quality of life of older people with dementia living in the community. Several limitations also need to be considered, including that the design did not include randomization, that the same physiotherapist performed the online assessments with the participant and carer as the physiotherapist supervising the exercise program (due to budget constraints), and that assessments were limited to using self-reported scales by the necessity of the entire program (assessments as well as intervention) being conducted online. Nonetheless, the results are promising and warrant further investigation in an adequately powered randomized controlled trial.

## 5. Conclusions

These study findings suggest that a telehealth exercise program delivered and progressed through intermittent videoconferencing by a physiotherapist, with carer supervision between the supervised online sessions over 12 weeks, is feasible and safe for older people with dementia living in the community. This program also showed improvements for people with dementia for a number of outcomes including physical activity level, disability, and quality of life. Additional strategies (e.g., tips to motivate carers and for carers to motivate people with dementia to continue with exercise) are needed in future programs to enhance adherence to the independent exercise program component (self-maintenance phase with carer supervision). A larger fully powered randomized controlled trial is necessary to clarify the effectiveness of the telehealth-delivered exercise program on this population.

## Figures and Tables

**Table 1:** Physiotherapist online visit and follow-up session schedule.

	Consent	Pre Assessment and Orientation	Exercise Intervention Duration (Week)												Post Assessment	Follow-up Assessment
Week			1	2	3	4	5	6	7	8	9	10	11	12	13	18
Physiotherapist online-visit sessions																
Check-up/support sessions																

Notes: Grey shaded area denotes consent and assessment periods of the program and grey block color is when the physiotherapist contacted the participants.

**Table 2:** Characteristics of the participants at baseline.

Characteristic	Participants with Dementia (n = 30)	Family Carers (n = 30)	Paid Carer (n = 10)
Age (Mean ± SD)	71.5 ± 8.8	42.6 ± 11.3	33.2 ± 11.7
Gender (N [%] female)	19 (63.3%)	24 (80%)	9 (90%)
Family carer relationship with person with dementia—n (%) - Spouse - Child - Grandchild	NA	3 (10%) 25 (83.3%) 2 (6.7%)	NA
Having paid carer—n (%)	10 (33.3%)	NA	NA
Living with family carer—n (%)	25 (83.3%)	NA	NA
Days spent (per-week) with older person with dementia (Mean ± SD)	NA	6.9 (0.4)	7 (0)
Dementia Type—n (%) - Alzheimer’s Disease - Vascular dementia - Frontotemporal - Parkinson’s dementia	22 (73.3%) 6 (20%) 1 (3.3%) 1 (3.3%)	NA	NA
Dementia duration (Mean ± SD) (year)	2.1 ± 1.2	NA	NA
T-MMSE score (Mean ± SD)	15.0 ± 3.3	NA	NA
Using walking aid (indoor)—n (%)	3 (10%)	NA	NA
Walking aid type (indoor) - Cane/stick - Pickup frame/rollator	2 (6.7%) 1 (3.3%)	NA	NA
Using walking aid (outdoor)—n (%)	4 (13.3%)	NA	NA
Walking aid type (outdoor) - Cane/stick - Pickup frame/rollator	2 (50%) 2 (50%)	NA	NA
Having fall in the past 12 months—n (%)	12 (40%)	NA	NA
Number of falls in the past 12 months—n (%) - 0 fall - 1 fall - 2 falls - 3 falls - 4 falls - 5 falls - >5 falls	18 (60%) 8 (26.7%) 1 (3.3%) 2 (6.7%) 0 (0%) 1 (3.3%) 0 (0%)	NA	NA
Other health condition—n (%) # - Arthritis - Respiratory condition - Parkinson’s Disease - Diabetes - Cardiac condition - Stroke - Osteoporosis - Back pain - Lower limb joint replacement - Other (e.g., vision problem)	6 (20%) 1 (3.3%) 4 (13.3%) 6 (20%) 3 (10%) 6 (20%) 3 (10%) 4 (13.3%) 1 (3.3%) 4 (13.3%)	NA	NA
Participants’ living location—n (%) - Major city - Inner regional - Outer regional - Remote/very remote	10 (33.3%) 11 (36.7%) 7 (23.3%) 2 (6.7%)	NA	NA

Notes: # Participants could have more than one other condition. Abbreviations: T-MMSE, Telephone Mini Mental State Examination; NA, not applicable.

**Table 3:** Number of exercises and number of exercise sessions undertaken and median adherence (%) to 12 weeks with physiotherapist’s supervision and 6-week self-maintenance (i.e., weeks 13–18) phase of the exercise program.

Baseline to 12 Weeks (60 Sessions; n = 30 Participants)			6-Week Self-Maintenance (i.e., Weeks 13–18) (30 Sessions; n = 26 Participants)		
Median Number of Exercises Undertaken Per Session (IQR [25, 75])	Median Number of Exercise Sessions Undertaken (IQR [25, 75])	Median Adherence % (IQR [25, 75])	Median Number of Exercises Undertaken Per Session (IQR [25, 75])	Median Number of Exercises Undertaken (IQR [25, 75])	Median Adherence % (IQR [25, 75])
7 (3) ^a^	50.5 (10.5)	84.1 (17.1)	6 (2) ^a^	20 (5)	66.7 (16.7)

Note: ^a^ The number of exercises recommended ranged between 8 to 10 exercises.

**Table 4:** One way ANOVA results for outcome measures across three time points—mean (SD), F value, *p* value and effect size ^c^.

Outcome Measures	Baseline	12 Weeks	18 Weeks	Group × Time ANOVA Result		Effect Size (Cohen’s d) for Significant Results Baseline—12 Weeks	Effect Size (Cohen’s d) for Significant Results Baseline—18 Weeks
				F	*p*		
PASE	55.1 (55.2)	94.8 (75.9) ^a^	96.0 (67.2) ^b^	3.66	0.030	0.60	0.66
**LLDFI–Function Component Total**	50.8 (14.4)	57.6 (16.4)	58.7 (15.6)	2.27	0.109	NS	NS
LLDFI–Function (Upper extremity domain) ^d^	54.6 (16.7)	63.7 (20.4)	63.7 (19.0)	2.33	0.104 ^d^	NS	NS
LLDFI–Function (Basic lower extremity domain) ^d^	63.1 (17.9)	77.9 (20.9) ^a^	79.5 (18.9) ^b^	6.61	0.002 ^d^	0.76	0.89
LLDFI–Function (Advanced lower extremity domain) ^d^	41.7 (18.9)	49.6 (19.4)	49.9 (19.0)	1.80	0.173 ^d^	NS	NS
**LLFDI–Disability Component–Frequency Dimension Total**	32.3 (10.4)	37.3 (9.4)	38.6 (11.1) ^b^	3.12	0.049	NS	0.58
LLFDI–Disability Component–Frequency Dimension–Social Role Domain ^e^	29.5 (13.6)	34.9 (10.9)	35.8 (10.8)	2.46	0.091 ^e^	NS	NS
LLFDI–Disability Component–Frequency Dimension–Personal Role Domain ^e^	28.5 (11.6)	36.4 (12.2) ^a^	36.7 (14.8) ^b^	3.87	0.024 ^e^	0.66	0.62
**LLFDI–Disability Component–Limitation Dimension Total**	47.0 (13.7)	53.1 (12.5)	55.3 (14.3) ^b^	3.02	0.054	NS	0.59
LLFDI–Disability Component–Limitation Dimension–Instrumental Role Domain ^e^	45.6 (16.3)	52.7 (12.1)	54.8 (13.2) ^b^	3.52	0.034 ^e^	NS	0.47
LLFDI–Disability Component–Limitation Dimension–Management Role Domain ^e^	44.2 (24.6)	53.6 (21.3)	55.2 (21.5)	2.11	0.128 ^e^	NS	NS
Vitality plus Scale	35.3 (5.8)	39.9 (6.1) ^a^	40.6 (5.3) ^b^	7.44	0.001	0.77	0.95
Icon-FES	31.1 (7.7)	28.0 (7.5)	28.0 (7.3)	1.78	0.175	NS	NS
8-item PACES	24.6 (4.2)	32.1 (1.9) ^a^	32.2 (2.6) ^b^	61.55	<0.001	2.33	2.17
QOL-AD (People with Dementia)	28.8 (5.2)	32.0 (5.3) ^a^	32.0 (5.1) ^b^	3.58	0.032	0.60	0.60
QOL-AD (Carer)	29.1 (5.3)	33.8 (4.8) ^a^	33.6 (4.4) ^b^	8.68	<0.001	0.91	0.90
NPI-Q–Severity	16.9 (6.9)	14.1 (7.1)	14.1 (6.5)	1.63	0.202	NS	NS
NPI-Q–Carer Distress	19.1 (11.4)	16.0 (9.2)	15.3 (9.6)	1.20	0.305	NS	NS
Zarit Caregiver Burden Scale	34.1 (13.8)	31.9 (13.7)	31.1 (12.3)	0.39	0.676	NS	NS

Notes: ^a^ significant difference baseline to 12 weeks, ^b^ significant difference baseline to 18 weeks, ^c^ significant difference 12 weeks to 18 weeks (No significant differences identified; therefore, no effect size column reported), ^d^ Bonferroni adjustment applied (*p* = 0.017, i.e., 0.05/3 items), and ^e^ Bonferroni adjustment applied (*p* = 0.025, i.e., 0.05/2 items). Abbreviations: PASE = Physical activity level; LLDFI = Late-life function and disability instrument; Icon-FES = Iconographical falls efficacy scale; 8-item PACES = 8-item physical activity enjoyment scale; QOL-AD = Quality of life in Alzheimer’s Disease; NPI-Q = Neuropsychiatric Inventory Questionnaire; NS = Not significant.

## Data Availability

The data presented in this study are available on request from the corresponding author. The data are not publicly available due to ethical restriction.
